# Suicide by Homeless Mental Health Patients in England: A 10-Year National Clinical Survey

**DOI:** 10.1192/bjo.2026.11123

**Published:** 2026-06-30

**Authors:** Sarah Jane Shackleton, Lana Bojanić, Louis Appleby

**Affiliations:** 1 University of Manchester, Manchester, United Kingdom; 2 National Confidential Inquiry into Suicide and Safety in Mental Health, Manchester, United Kingdom

## Abstract

**Aims:**

Suicide is a significant cause of death worldwide and is prevalent among homeless individuals. However, aspects of suicide in homeless mental health patients remain under-researched. This study aims to analyse socioeconomic, clinical, and service-related characteristics of homeless mental health patients who died by suicide in England between 2012 and 2022, with a focus on age group and ethnic background.

**Methods:**

This retrospective cohort cross-correlational study uses data from the National Confidential Inquiry into Suicide and Safety in Mental Health (NCISH). Homelessness was classified as having ‘no fixed abode’. Comparative analyses, including multivariable logistic regression, were conducted between homeless (n=324) and non-homeless patients (n=12,605). Subgroup analyses were performed within the homeless group based on age group (under 45 years and over 45 years) and ethnic background (Ethnic Minority background and White-British).

**Results:**

Homeless patients were more likely to be male, younger and socioeconomically disadvantaged, to have dual diagnoses of substance dependence, adjustment disorders and histories of substance misuse, self-harm and service disengagement. They were more likely to die within 24 hours of last service contact. Younger homeless patients displayed higher prevalence of substance misuse and self-harm, and for self-harm leading to service contact. Older homeless patients more often faced recent financial difficulties. Homeless patients from ethnic minority backgrounds had a higher prevalence of affective disorders, immigration-related vulnerability and a concerningly lower median age of death.

**Conclusion:**

Suicide in homeless mental health patients is associated with complex socioeconomic and clinical vulnerabilities. Our findings emphasise the need for integrated, culturally sensitive and targeted suicide prevention strategies.

